# Long-Term Toxicity Study of Topical Administration of a Highly-Stable rh-aFGF Carbomer 940 Hydrogel in a Rabbit Skin Wound Model

**DOI:** 10.3389/fphar.2020.00058

**Published:** 2020-02-21

**Authors:** Li Zhang, Tongzhou Huang, Jianing Bi, Yingying Zheng, Chao Lu, Qi Hui, Xiaojie Wang, Xiaohua Lin

**Affiliations:** ^1^ The Department of Dermatology of the First Affiliated Hospital of Wenzhou Medical University, Wenzhou, China; ^2^ School of Pharmacy of Wenzhou Medical University, Wenzhou, China; ^3^ Engineering Laboratory of Zhejiang Province for Pharmaceutical Development of Growth Factors, Biomedical Collaborative Innovation Center of Wenzhou, Wenzhou, China

**Keywords:** rh-aFGF, carbomer 940 hydrogel, long-term toxicity, immunotoxicity, neutralization antibody

## Abstract

We developed a highly stable recombinant human acidic fibroblast growth factor (rh-aFGF) carbomer 940 hydrogel for wound healing. This study aimed to reveal toxicity target organs and the toxicity dose-response in the long-term administration of rh-aFGF carbomer 940 hydrogel in a rabbit skin wound model. New Zealand rabbits were topically administrated rh-aFGF carbomer 940 hydrogel at a daily dose of 900 IU/cm^2^, 1,800 IU/cm^2^, and 3,600 IU/cm^2^ for 28 days. Lyophilized rh-aFGF agent was used as the positive control group. General behavior, serum chemistry, skin irritation, immunogenicity, immunotoxicity, and histopathology were analyzed at designated time points. Results revealed that food intake, body weight, body temperature, heart rate, and eye examinations were all normal, suggesting no obvious toxicity induced by the rh-aFGF hydrogel. Medium and high dose rh-aFGF hydrogel groups and the positive control group displayed increased cell numbers in the local lymph nodes near the site of administration, likely caused mesenteric lymph node follicular hyperplasia, and this observation was alleviated after 14 days of recovery. Immunogenicity studies demonstrated that the serum antibody titer against rh-aFGF increased with the duration and number of drug applications but were not neutralization antibodies. After administration stopped, antibody titer decreased and disappeared in some mice. In summary, the safe dose for long-term administration of rh-aFGF carbomer 940 hydrogel for persistently damaged skin was 900 IU/cm^2^, which is 10 times that of the proposed clinical dosing.

## Introduction

Human acidic fibroblast growth factor (rh-aFGF), a member of the fibroblast growth factor family (FGF) (Beenken and Mohammadi, 2009; Li, 2019), is a potential therapeutic option for wound healing (Mellin et al., 1995; Wu et al., 2016). The preliminary mechanisms through which aFGF promotes the wound healing include aFGF-stimulated secretion of TGF-β and PCNA proteins from the damaged skin and the promotion of capillary and fibroblast proliferation (Xie et al., 2011). Our group previously developed a lyophilized rh-aFGF, which was approved by the Chinese Food and Drug Administration in 2006 for the treatment of deep, partial-thickness burns and skin graft donor sites (Ma et al., 2007; Hui et al., 2018a). This marketed rh-aFGF is a topical lyophilization agent, applied in the form of a spray. The inconvenience of this spray preparation included a short residence time on the skin and the requirement of frequent administration. Carbomer 940 hydrogel is a high molecular weight material with good biocompatibility that does not cause skin irritation (Sahoo et al., 2016; Cui et al., 2017; Hayati et al., 2018). Carbomer 940 hydrogel also provided favorable conditions for wound healing due to its high moisture retention, sustained drug release, and good adhesion (Wu et al., 2016). Based on the above advantages of carbomer 940, our group developed an rh-aFGF carbomer 940 hydrogel that demonstrated notable healing efficacy in GK rat diabetic skin ulcers and whole-layer skin resection models, and was superior to the rh-aFGF solution (Hui et al., 2018b). SEM imaging showed that the rh-aFGF-carbomer hydrogel contained slight 3D network structures, which allowed the diffusion of growth factors. This may be one reason why the rh-aFGF carbomer hydrogen was superior in promoting wound healing compared to the solution. Our previous *in vivo* studies also revealed that the rh-aFGF carbomer hydrogel significantly promoted the proliferation of fibroblasts and enhanced angiogenesis (Hui et al., 2018b). The course of treatment is generally long when using rh-aFGF to treat chronic ulcers (Xie et al., 2011; Wu et al., 2016), and early pharmacokinetic studies demonstrated that when applied to the damaged skin of rabbits, some rh-aFGF was absorbed into the systemic circulation. In addition, the half-life of the drug-loaded hydrogel was much longer than the lyophilization-prepared rh-aFGF (Huang et al., 2011; He et al., 2019). Therefore, understanding the long-term safety of rh-aFGF hydrogel is of great significance for its clinical application (Nilsson et al., 2012; Duenas-Gonzalez et al., 2014). The purpose of this study was to examine skin irritation and the long-term systemic safety of rh-aFGF hydrogel in a New Zealand white rabbit skin damage model, exceeding 10 times the clinical dose and with continuous treatment for 28 days, to determine possible adverse reactions.

## Materials and Methods

### Materials

Rh-aFGF carbomer 940 hydrogel was prepared as previously described (Hui et al., 2018b) (lot numbers: 20121101, 20121102, and 20121103, and sizes were 225,000 IU/10 g/vial, 450,000 IU/10 g/vial, and 900,000 IU/10 g/vial, respectively, pH 6.9); Negative control: Carbomer 940 hydrogel (product lot number: 20121104, size: 10 g/vial, pH 6.9) stored at 4°C. Positive control: lyophilized rh-aFGF for topical application purchased from Shanghai Wanxin Pharmaceutical Limited (Shanghai, China, product lot numbers: 20121202 and 20121203, size: 25,000 U/vial, pH 7.0) stored at 2–8°C, away from light.

### Animals

New Zealand rabbits (20 males and 20 females, body weight 1.88–2.74 kg, aged 120–160 days) were purchased from Shanghai Shengwang Experimental Animal Limited (Shanghai, China). Experiments were conducted at the GLP Laboratory of the Suzhou Xishan Drug Safety Evaluation Research Co., Ltd. (Suzhou, China). Protocols were approved by the Institutional Animal Care and Use Committee (IACUC), and animals were cared for per guidelines for the Care and Use of Laboratory Animals. Animals were kept at a temperature of 20.1–23.2°C and a relative humidity of 42.0%–68.2%. Each rabbit was kept in a single cage with dimensions of 42×50×35 cm^3^ and provided with standard rabbit feed and experimental animal drinking water.

### Dose Design and Groups

Dose design is very critical for evaluating systemic and local irritation from the rh-aFGF hydrogel, as the toxicity of transdermal administration is related to the area of administration. In a clinical setting, the maximum possible dose is 10% of the body surface area, and 10% of the body surface area of a typical 2 kg New Zealand rabbit is approximately150 cm^2^. Therefore, the administration area of our long-term toxicity study was 150 cm^2^. The clinically recommended dose of the rh-aFGF-loaded hydrogel is 90 IU/cm^2^. The lowest dose was chosen to be 10 times the effective dose, or 900 IU/cm^2^. The ratio of the low, medium, and high-dose groups was 1:2:4.

New Zealand rabbits (N = 40) were randomly divided into 5 groups: negative control group, rh-aFGF gel groups (low, medium, and high dose), and rh-aFGF lyophilization positive control group. There were 8 animals in each group, half male and half female. Hair on both sides of the back was removed, and a “#”-shaped cut was made using sterilized needles twice a week. The rh-aFGF-loaded hydrogel (3 g) was weighed, placed on 10 cm×7.5 cm sterile weight paper, spread evenly, and applied to one side of the damaged rabbit skin. The same procedure was done on the other side of the skin. The negative and positive control groups underwent the same procedure. All sites of administration were covered with sterile gauze and secured with medical tape. Dosing was administrated for 6 h once daily for 28 days, and then the animals recovered for 14 days.

### General Vital Sign Observations

Animals were observed for general behavior, food intake, and defecation status before daily dosing. Rabbits were weighed weekly, and body temperature was monitored 0.5 h, 1 h, 2 h, and 3 h after drug administration. The eyelids, conjunctiva, cornea, iris, pupil, and fundus of the rabbits were examined using an XQ-I ophthalmoscope (Tecente, Jiangsu, China) on days 0 and 28 of dosing and 14 days after the final dose.

### Electrocardiogram

Electrocardiogram profiles were checked on days 0 and 28 after the first dose as well as 14 days after the last dose using an ECG-6511 Electrocardiograph (Shanghai Optoelectronic Medical Electronics Co, Shanghai, China). The following parameters were measured: heart rate, P wave, R wave, T wave, PR interval, QRS interval, QT interval, and ST segment.

### Serum Chemistry

Serum chemistry was performed at three timepoints, on days 0 and 28 of dosing and 14 days after the final dose. Blood (3 ml) was collected from the hind limbs of the animals using a disposable vacuum negative pressure tube, and coagulation function was analyzed *via* an automatic blood coagulation analyzer (DIAGNOSTICA STAGO STA-R Evolution, USA). Blood cell sorting and counting of white blood cells (WBC), red blood cells (RBC), and platelets (PLT) were conducted using a blood analyzer (SYSMEX CORPORATION XT-2000iV, USA) to evaluate whether the inflammatory reaction of the animal and the drug was toxic to the hematopoietic system. Analysis of serum chemistry indicators such as total bilirubin (TBIL), alanine aminotransferase (ALT), aspartate aminotransferase (AST), and alkaline phosphatase (ALP) was performed using an automated biochemical analyzer (Abbott ARCHITECT c8000, USA), mainly to assess liver function in the animals.

### H&E Staining

On day 28 of dosing and on day 14 post-dosing, 4 animals in each group were injected with 60 mg/kg of sodium pentobarbital *via* the ear vein, and the femoral artery was exsanguinated and dissected. The epididymis, ovaries, adrenal glands, thymus, uterus, testes, heart, liver, spleen, lungs, kidneys, and brain were weighed, and the absolute weight of each organ was recorded. The ratio of the weight of each organ to that of the brain was calculated. All aforementioned organs, skin from the site of administration, the subscapular lymph nodes (both sides), every component of the digestive tract, and the accessory glands were fixed with 10% formalin, dehydrated, and then embedded in paraffin. Sections (5 μm) were prepared and stained with hematoxylin-eosin, and a thorough pathological examination was performed using a Nikon ECLIPSE Ti microscope (Nikon, Tokyo, Japan).

### Total Antibodies Measured by ELISA

On days 0, 14, 21, and 28 of dosing, and 14 days after the last dose, total antibodies against rh-aFGF in the rabbit serum were measured by indirect ELISA. Absorbance was measured at 450 nm and 630 nm using a Bio-TekELx800 universal microplate reader (Bio-Rad, CA, USA), according to the instructions of the manufacturer. The cutoff was set at 2.1-fold of the average measured values of the pre-dose animals. If the absorbance value of the serum sample after administration was greater than the cutoff, it was deemed positive.

### Neutralization Antibodies Measured by MTT

NIH 3T3 cells in the log growth phase were collected and seeded into 96-well plates at a concentration of 7,000–9,000 cells per well. After incubation for 48 h, filter-sterilized rabbit serum samples diluted 2,000-fold in DMEM medium containing 10 ng/ml rh-aFGF were added, and a serum sample of the pre-dose animal was used as a control. After incubating at 37°C and 5% carbon dioxide for 36–48 h, 20 μl of MTT solution was added to each well. After another 4 h, the supernatants were discarded, and 100 μl of dimethyl sulfoxide was added to each well. After proper mixing, the absorbance of each well was measured at 570 nm using a Bio-TekELx800 microplate reader (Bio-Rad, CA, USA). The ratio of absorbance of the sample and the control group was used to determine cell viability. When cell viability was less than 50%, the presence of neutralization antibodies was considered positive.

### Evaluation of Immunotoxicity

Immunotoxicity assessment was conducted based on white blood cell count and classification, albumin, immunoglobulin, ratio of albumin to immunoglobulin, weight of the spleen and thymus, visceral/body weight ratio, visceral/brain ratio, anatomical results of the bone marrow, spleen, thymus, mesenteric lymph nodes and subscapular lymph nodes (both sides), and histopathological findings.

### Statistical Analysis

Statistical analysis was conducted using SPSS 13.0 software. Data were expressed as mean ± standard deviation (SD), and *P* < 0.05 indicated statistical significance.

## Results

### General Vital Sign Observations

**Figure 1:**
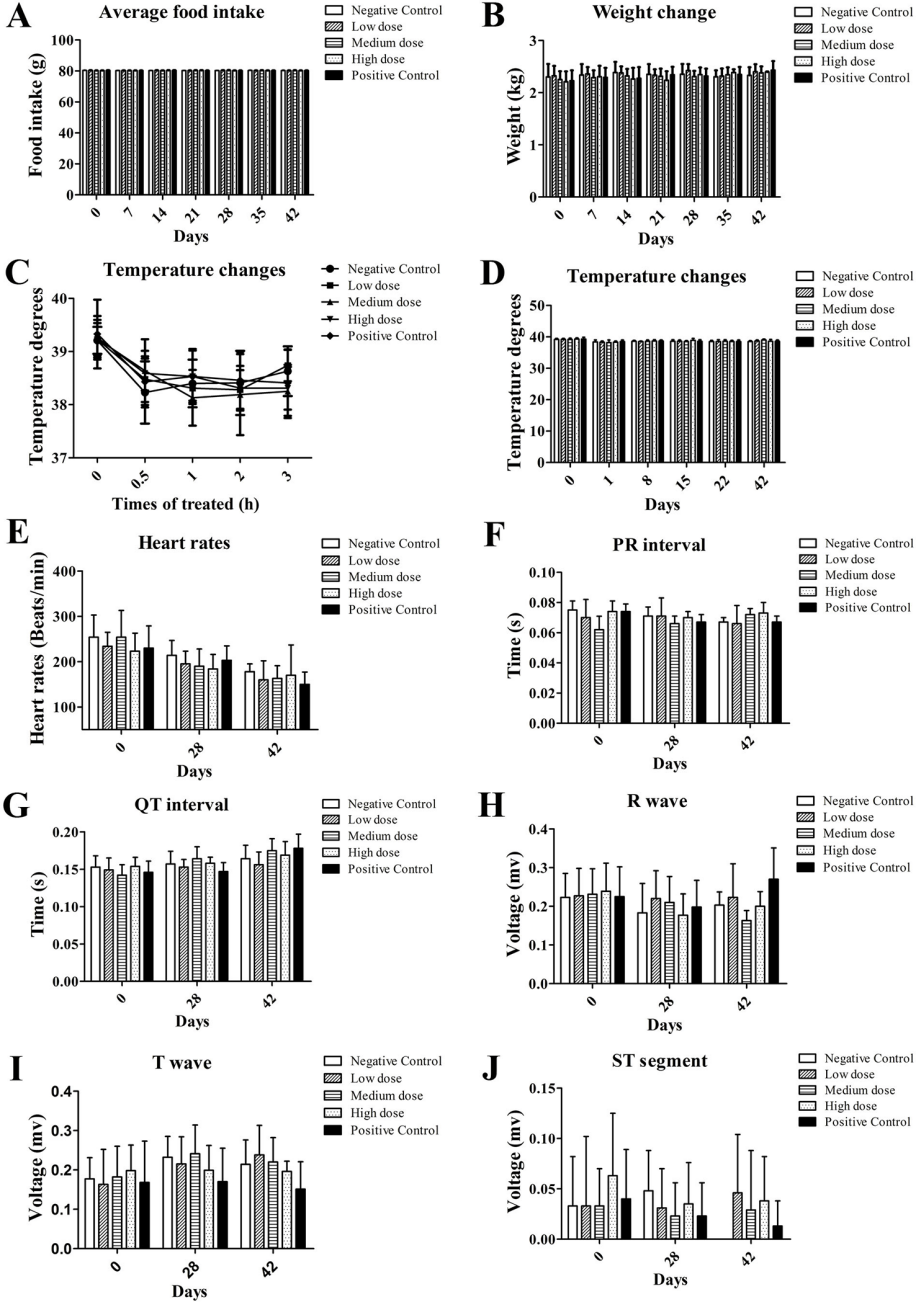
General vital signs and electrocardiograms after topical administration of rh-aFGF hydrogel. **(A)** Food intake. **(B)** Body weight. **(C, D)** Body temperature. **(E)** Heart rate. **(F–J)** ECG, n = 8 at 28 days and n = 4 at 42 days.

Sudden death or signs of dying were not observed in all dose groups during the 28-day administration. Other than red and swollen skin at the wound site due to the needle scratch, no obvious adverse reactions were observed. There was no significant difference in feeding behavior and body weight between the dose groups and the control group at the corresponding time points (**Figures 1A, B**, *P* > 0.05). There was no difference in body temperature between the negative control, the rh-aFGF hydrogel treatment, and the positive control group during the administration period (**Figures 1C, D**, *P* > 0.05).

### Electrocardiogram and Serum Chemistry

The heart rate, P-R interval, Q-T interval, R wave, T wave, and ST segment of the rabbits in each dose group showed no significant changes compared to the negative and positive control groups (**Figures 1E–J**, *P* > 0.05). Similarly, on the 28th day of administration and at the end of the recovery period, blood coagulation index and serum chemistry in each dose group were not significantly different from the negative and positive control groups (data not shown).

### Evaluation of Vital Organs

**Figure 2:**
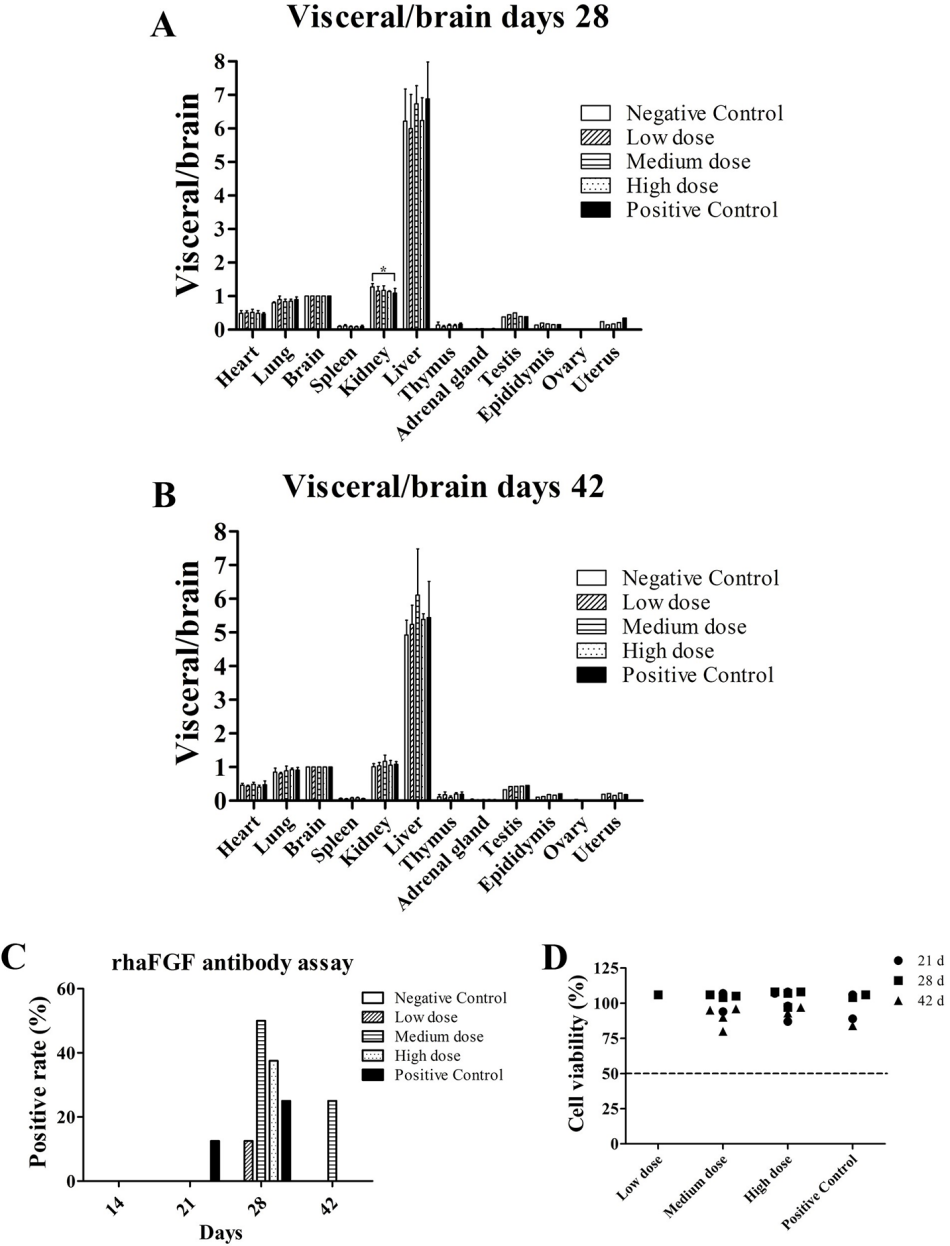
Visceral/brain ratio and anti-rh-aFGF antibodies. **(A, B)** Visceral/brain ratio at Day 28 and Day 42, n = 4. **(C)** Total anti-rh-aFGF antibody in rabbit serum. **(D)** Neutralization antibody detection *via* MTT viability assay. *P ≤ 0.05, n = 8 at 28 days and n = 4 at 42 days.

At the end of the rh-aFGF hydrogel treatment period and the post-recovery phase, no obvious pathological findings such as congestion, hemorrhage, necrosis, adhesion, abnormal mass formation, or organ dysplasia were observed upon visual examination. No organ deformation or developmental abnormality was seen. In terms of organ weight, there was no difference in the ratio of visceral versus whole body weight, visceral versus brain weight, or kidney weight between the treatment and control groups at the same time points. During the recovery phase, all treatment groups were comparable, as depicted in **Figures 2A, B**.

### rH-aFGF Immunogenicity Evaluation

Rabbit blood samples contained anti-rh-aFGF antibodies in each dose group and the positive control group after skin administration, and the antibody titer increased with the time and duration of treatment. After stopping the drug, the antibody titer decreased, and in some cases, disappeared (**Figure 2C**). Results of the neutralizing antibody assay using the sera of the rabbits showed that the viability of NIH 3T3 cells was > 50% (**Figure 2D**), indicating that no significant amount of neutralizing antibody was produced.

### Pathological Detection of Skin Tissue

**Figure 3:**
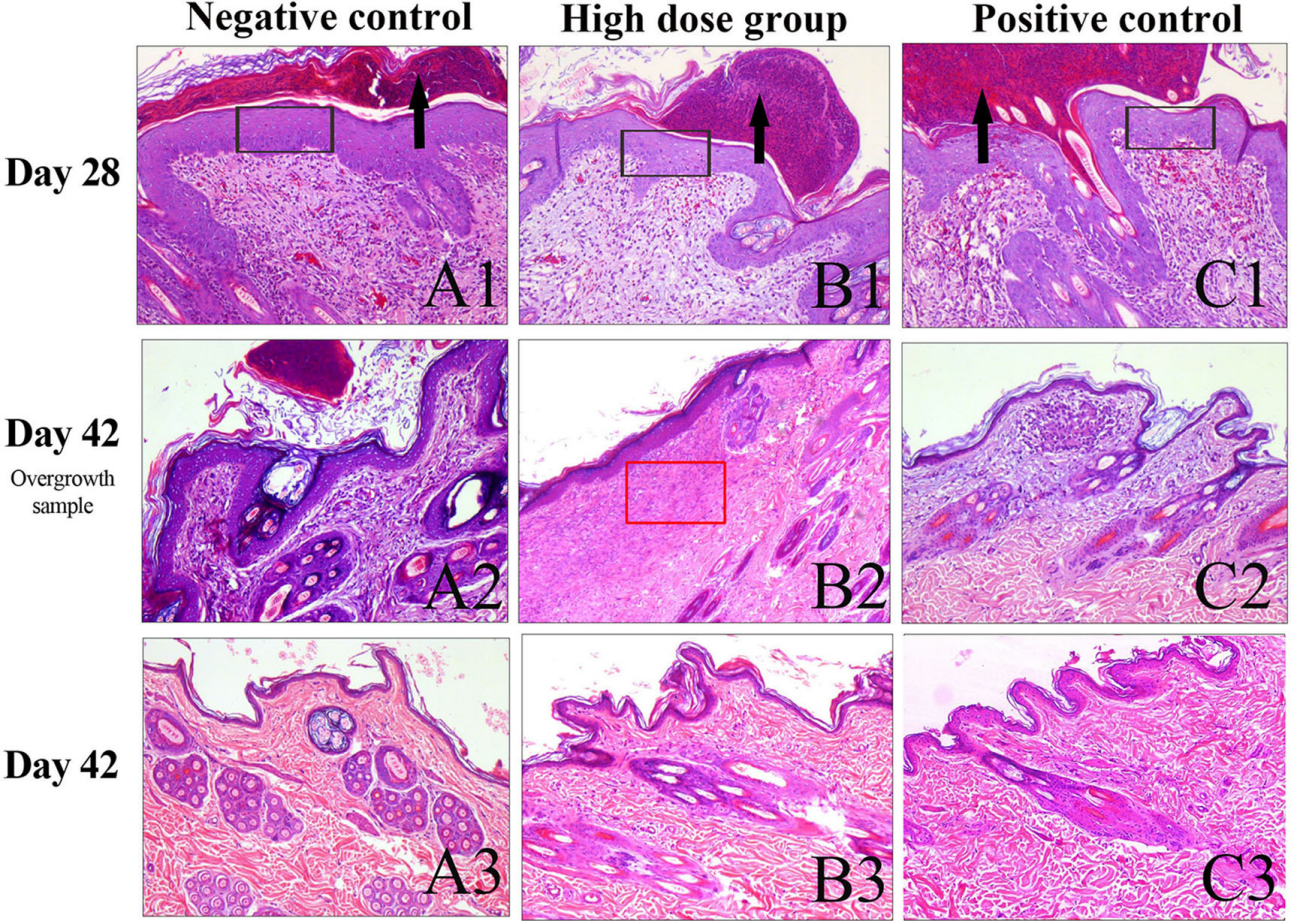
Histopathology of the skin observed with H&E staining. **(A)** Skin samples around the site of administration at day 28. **(B)** Skin sample around the site of administration with slight overgrowth among the experimental groups on day 42. **(C)** Skin samples around the site of administration had recovered substantially on day 42. The black arrow indicates the scab. The black rectangle indicates epidermal thickening. The red rectangle indicates collagen deposition and fibroplasia. Scale bars:100 μm.

On the 28th day of administration, epidermal thickening, the formation of ecdysis, dermal layer congestion, hemorrhage, a small amount of inflammatory cell infiltration, and fibrous tissue hyperplasia were observed around the skin near the site of administration in all groups (**Figures 3A1–C1**). At the end of the recovery phase, the skin of most of the rabbits in the negative control group, the high-dose group, and the positive control group had recovered at the site of administration (**Figures 3B, C**).

### Immunotoxicity Evaluation

**Figure 4:**
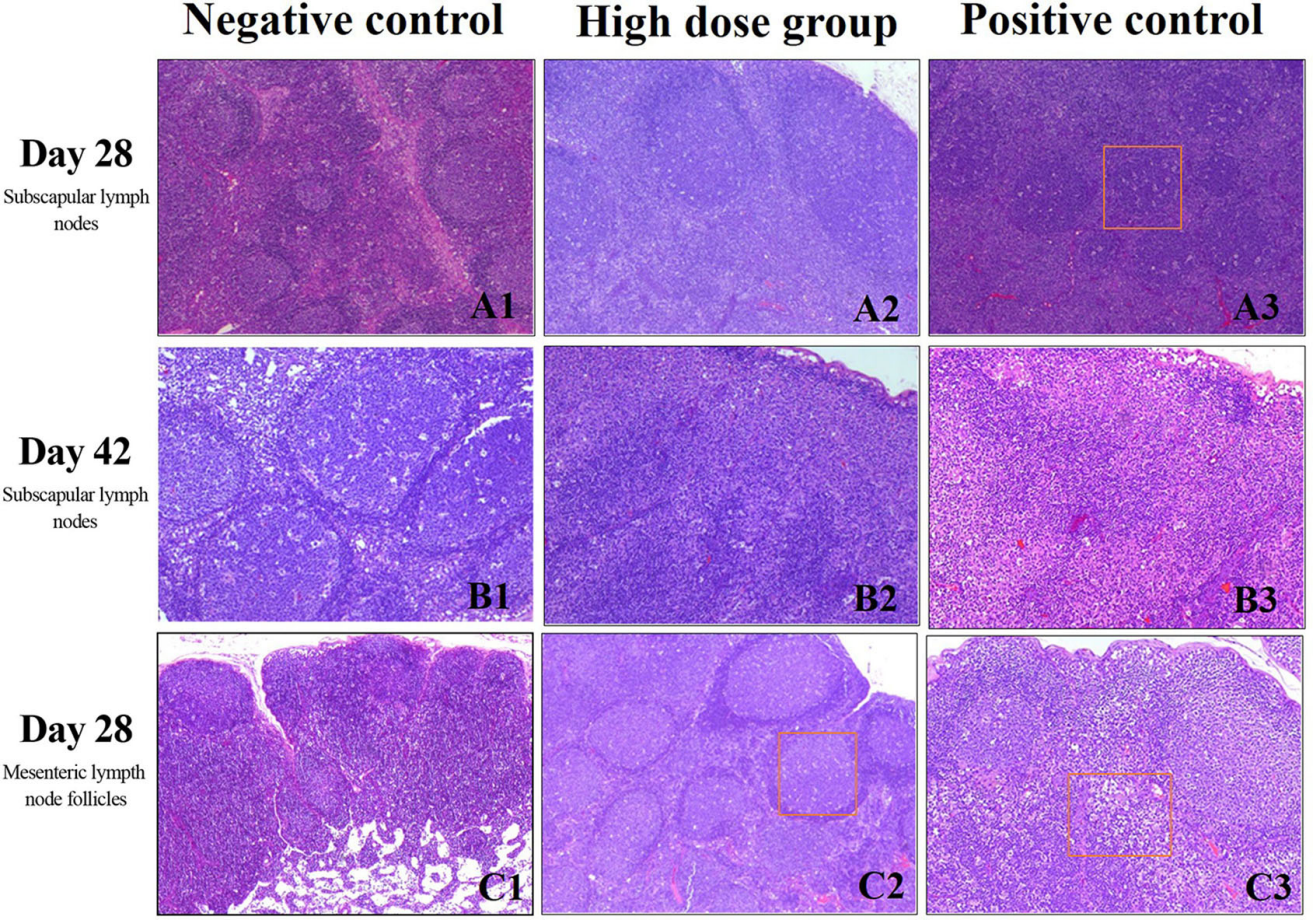
Histopathology of lymph nodes evaluated with H&E staining. **(A)** Subscapular lymph nodes at day 28, scale bars: 40 μm **(B)** Subscapular lymph nodes at day 42, scale bars:100 μm **(C)** Mesenteric lymph node follicles at day 28 day, scale bars: 4 0 μm. Rectangular box indicates “starry sky” appearance.

At the end of treatment and during the recovery phase, animals in all groups had enlarged lymphoid follicles in the subscapular lymph nodes as well as an increase in the number of follicles, with expanded germinal centers and an obvious “starry sky” appearance. At the end of the recovery phase, most of the animals in the high-dose and positive control group had increased cell numbers in the subscapular lymph nodes (**Figures 4A, B**). Mesenteric lymph node follicles appeared enlarged, had increased in number, and had expanded germinal centers and sometimes a “starry sky” appearance. This was mostly seen in the high dose and positive control groups (**Figure 4C**) and was alleviated after the recovery phase.

## Discussion

It has been well-documented that aFGF plays an important role in wound healing (Zheng et al., 2015; Hota et al., 2018; Wang et al., 2018; Kawano et al., 2019). Compared to the lyophilized rh-aFGF currently on the market, the rh-aFGF-loaded carbomer hydrogel has the advantage of increased drug intake and enhanced bioavailability, providing the wound with a “wet healing environment” and avoiding secondary contamination (Hui et al., 2018b). However, aFGF can enter the systemic circulation through the wounded skin, and potential toxicity of the polymer biomaterial itself exists (Guadagno and Miranda, 2017; Huang et al., 2019). Therefore, the long-term repeated administration of the rh-aFGF carbomer 940 hydrogel in the treatment of refractory ulcers should be investigated to shed light on the dose/toxicity relationship and to identify potential target tissues or organs. This information will provide a basis for human clinical studies.

In the present study, we utilized the New Zealand white rabbit back scratch model and dosed rh-aFGF hydrogel at equal to or higher than 10 times the clinical dose repeatedly for 28 days to observe the time course of toxicity, identify potential target organs or tissues, and determine a safe dose range. There were no animal deaths due to drug-related toxicity after long-term administration, and no target organs or tissues for toxicity found after general observation of the animals and examination of various systems such as the circulatory, respiratory, digestive, and cardiovascular systems. However, pathological observation of the skin tissue revealed epidermal thickening, ecdysis formation, dermal layer congestion, hemorrhage, a small amount of inflammatory cell infiltration, and fibrous tissue hyperplasia in every dose group at the end of the administration period. We believe this was mainly caused by mechanical irritation because some animals in the negative control group developed local abscesses and dermal structural destruction due to deep wound and local infection. However, at the end of the recovery period, the skin of most of the animals in the negative control, high-dose, and positive control groups recovered substantially. Only a single animal displayed slight thickening of the epidermis, molt and mildly increased inflammatory cell infiltration and collagen deposition and fibroplasia in rh-aFGF high dose group (**Figure 3B2**). Taken together, the rabbit skin in each group recovered after the dosing ended, and the rh-aFGF carbomer hydrogel induced no long-term irritation or hyperplasia at the site of administration. As a wound repair growth factor, aFGF can upregulate the expression of collagen fibers and muscle fibers, resulting in enhanced tissue remodeling (Zheng et al., 2015). When developing this compound as a drug, we should also consider whether excessive tissue repair after long-term repeated application would occur, resulting in over-expression of local collagen around the wound and scar formation. Our results demonstrated that the high-dose, repeated administration process did not cause excessive repair of the tissue or scar formation.

During the immunotoxicity study, we found that at the end of the drug administration period and the recovery phase, there was unilateral or bilateral lymphoid follicle enlargement, an increase in follicle numbers, germinal center enlargement, and a “starry sky” phenomenon in the subscapular lymph nodes. The above lymph node changes were only occasionally seen in the negative control group, while the increase in cell numbers in the subscapular lymph nodes was only seen in the high-dose and positive control groups. In the pathological analysis of mesenteric lymphoid tissue, we found that some animals in the drug-administered group had increased mesenteric lymph node follicles, increased cell numbers, and enlarged germinal centers, although this phenomenon had no significant dose-response relationship and was mainly seen in the medium- and high-dose and positive control groups. We hypothesize that because the rh-aFGF was from humans and has a molecular weight of 16 kDa, it could be considered a foreign macromolecule in New Zealand rabbits and thus elicited an immune response (Adel-Patient et al., 2018). Additionally, previous studies have shown that aFGF can activate lymphocytes and other immune cells by regulating macrophages and participating in inflammatory and cellular immune responses (Ichinose et al., 1998).

During the immunogenicity study, we found that the serum total antibody level against rh-aFGF increased, but this did not appear to be neutralizing. In addition, the antibody titer decreased or even disappeared after stopping the drug. Therefore, the production of the antibody did not reduce the effective concentration of rh-aFGF on the wound and did not cause other immune reactions.

In summary, rh-aFGF carbomer 940 hydrogel showed no obvious toxicity to the target organs and tissues during long-term repeated treatment in a New Zealand white rabbit skin damage model. Considering the immunotoxicity in the medium, high-dose, and positive control groups, the safe dose for long-term administration of the rh-aFGF carbomer 940 hydrogel for persistently damaged skin was determined to be 900 IU/cm2, which is 10 times that of currently proposed clinical dosing. However, some immunogenicity was observed in this study, so close attention should be paid in future clinical trials.

## Data Availability Statement

The datasets generated for this study are available on request to the corresponding authors.

## Ethics Statement

The animal study protocols were approved by the Institutional Animal Care and Use Committee (IACUC) and animals were cared for per the guidelines for the care and use of laboratory animals.

## Author Contributions

LZ, TH, and JB carried out the studies and collected data. YZ and CL performed the statistical analysis and participated in the study design. QH participated in the acquisition and analysis of data. XW and XL designed the study and wrote the manuscript. All authors have read and approved the final manuscript.

## Funding

The study was supported by the National Natural Science Foundation of China (No. 81601695); Science and Technology Project of Wenzhou (No. Y20180150).

## Conflict of Interest

The authors declare that the research was conducted in the absence of any commercial or financial relationships that could be construed as a potential conflict of interest.

## Abbreviations

rh-aFGF_135_, recombinant human acid fibroblast growth factor_135_; WBC, white blood cells; RBC, red blood cells; PLT, platelets; TBIL, total bilirubin; ALT, alanine aminotransferase; AST, aspartate aminotransferase; ALP, alkaline phosphatase.
